# Quantification of muco-obstructive lung disease variability in mice via laboratory X-ray velocimetry

**DOI:** 10.1038/s41598-020-67633-y

**Published:** 2020-07-02

**Authors:** Freda Werdiger, Martin Donnelley, Stephen Dubsky, Rhiannon P. Murrie, Richard P. Carnibella, Chaminda R. Samarage, Ying Y. How, Graeme R. Zosky, Andreas Fouras, David W. Parsons, Kaye S. Morgan

**Affiliations:** 1 Department of Mechanical and Aerospace Engineering, Monash University, Melbourne, Australia; 2 Robinson Research Institute and Adelaide Medical School, University of Adelaide, Adelaide, Australia; 3 Women’s and Children’s Hospital, North Adelaide, Australia; 4 4Dx Limited, Melbourne, Australia; 5 School of Physics and Astronomy, Monash University, Melbourne, Australia; 6 School of Medicine, University of Tasmania, Hobart, Australia; 7 Menzies Institute for Medical Research, University of Tasmania, Hobart, Australia

**Keywords:** X-ray tomography, Computational models, Data mining, Respiration, Biomedical engineering

## Abstract

To effectively diagnose, monitor and treat respiratory disease clinicians should be able to accurately assess the spatial distribution of airflow across the fine structure of lung. This capability would enable any decline or improvement in health to be located and measured, allowing improved treatment options to be designed. Current lung function assessment methods have many limitations, including the inability to accurately localise the origin of global changes within the lung. However, X-ray velocimetry (XV) has recently been demonstrated to be a sophisticated and non-invasive lung function measurement tool that is able to display the full dynamics of airflow throughout the lung over the natural breathing cycle. In this study we present two developments in XV analysis. Firstly, we show the ability of laboratory-based XV to detect the patchy nature of cystic fibrosis (CF)-like disease in β-ENaC mice. Secondly, we present a technique for numerical quantification of CF-like disease in mice that can delineate between two major modes of disease symptoms. We propose this analytical model as a simple, easy-to-interpret approach, and one capable of being readily applied to large quantities of data generated in XV imaging. Together these advances show the power of XV for assessing local airflow changes. We propose that XV should be considered as a novel lung function measurement tool for lung therapeutics development in small animal models, for CF and for other muco-obstructive diseases.

## Introduction

Cystic fibrosis (CF) is a progressive, chronic and debilitating genetic disease caused by mutations in the CF Transmembrane-conductance Regulator (*CFTR*) gene. Unrelenting CF airway disease begins early in infancy and produces a steady deterioration in quality of life, ultimately leading to premature death. Effective lung health assessment tools must capture the patchy nature of muco-obstructive lung diseases such as cystic fibrosis, and this is particularly important during the early stages of the disease when local treatments could be applied to prevent disease progression.

Assessments of overall lung health in humans and animals are typically made using lung *function* tests that screen for abnormalities by measuring the flow of gas in the airways. Spirometry is the most common tool for assessing lung *function*, however it measures global airflow at the mouth. This means that parameters such as FEV_1_ are a single, global measure of the health of the entire lung, are effort-dependent, and provide no information about the location of muco-obstructive disease. Multiple breath wash-in or wash-out techniques that measure the lung-clearance index (LCI) are sensitive to some changes in CF disease as they reflect the health of both the small and large airways^1, 2^, but can only provide limited regional airflow information. LCI is rarely reported in laboratory animal studies. The forced oscillation technique (FOT) measures the resistive properties of the respiratory system and has been used in obstructive lung disease assessment, although the results can be difficult to interpret. Furthermore it suffers from the same problems as FEV_1_ with respect to lumping regional information into single whole-lung parameters^3^. These kinds of techniques that are used clinically can also be applied in animals using current gold-standard tools such as the flexiVent (Scireq, Canada), to measure some of the effects of pharmaceutical and genetic therapeutics in animal models^4^. Regardless of the measurements that can be provided by these common measures of lung function, they cannot accurately localise the changes in airflow that are caused by structural abnormalities within the lung.

To identify structural lung disease, image-based assessments such as CT and MRI are commonly used in humans and animal models. CT provides excellent *structural* information, and can therefore be used to monitor for structural abnormalities from disease progression, or morphological changes produced by new drug therapies. However, preventative treatment programs should ideally be able to intervene before disease establishes and progresses to the point at which it produces structural changes. Similarly, therapeutics assessments should be able to detect early functional changes^4^. MRI has the advantage of being able to simultaneously observe both lung *function* and *structure* without exposing the subject to ionising radiation. However, progress in MRI research has lagged behind x-ray-based methods, most likely because spatial resolution is poor and the properties of the lung—particularly the low proton density, present since the lung is comprised primarily of air—make it less appropriate for MRI^5^. Nonetheless, recent innovations such as hyperpolarised gas and ultrashort echo time imaging continue to advance human chest MRI research^6, 7^.

Methods that attempt to infer lung *function* from CT and MRI images have previously been reported in humans and animals. Spirometry-guided CT for volume control during imaging has potential benefits but has not been implemented on a large scale and requires extensive patient training to administer^5^. Scoring systems that use software analyses to validate outcome measures from chest CTs have been developed, including the PRAGMA-CF assessment protocol for monitoring early-stage CF in children^8^. These methods must still look to repeatability and standardization of results, as well as restrictions associated with radiation dose from repeated chest CTs^9^. Other developments that show promise include registration-based techniques for measuring lung aeration^10–12^, and the use of contrast agents to render lung content directly visible^13, 14^. Tracking X-ray microscopy (TrXM) takes advantage of a sophisticated synchrotron-based X-ray imaging system to directly image mouse alveoli during respiration^15^.

Despite the availability of techniques that assess either lung *function* or lung *structure*, none of these are able to simultaneously identify the origin of changes in function, and evaluate their heterogeneity. Abnormal lung motion during breathing has been demonstrated to be an indicator of disease^16^. Our previous research has developed a method that can rapidly capture the motion of the natural breathing cycle at a high spatial and temporal resolution, without the use of a contrast agent. To do this, propagation-based phase-contrast X-ray imaging (PCXI) was utilised. Since PCXI does not rely solely on absorption of X-rays by matter—but rather the diffraction of rays at material interfaces enhanced by the propagation of x-rays through free space—it can reduce the radiation dose and health risk associated with conventional chest CT scans^17–19^. PCXI can be combined with tomography to create detailed three-dimensional reconstructions of the fine structures in the lungs^20–23^. Using multiple PCXI images acquired throughout the respiratory cycle, Fouras et al.^16^ applied particle-image velocimetry to determine the speed and direction of lung motion in three dimensions throughout the respiratory cycle. The resulting regional maps of lung tissue motion can be used to detect subtle and non-uniform lung disease^24–26^. This high-speed PCXI acquisition and post-processing analysis is termed *X-ray velocimetry* (XV). The key difference between standard structural imaging modalities and XV is that XV assesses the dynamics of the lung tissue movement throughout the breath in order to extract measures of tissue expansion. The result is a detailed ventilation map of the lung, which non-invasively enables the volume of air that flows through each branch of the lung tree to be calculated^21^.

The potential value of XV for inferring lung *function* is shown by the characterisation of CF-like lung disease in small laboratory animals at high resolution using a synchrotron-based X-ray source^24^, where the spatial and temporal variability in airflow throughout the lung was assessed. Recently, Murrie et al*. *reported the proof-of-principle translation of XV to a laboratory-based source^25^. They showed that despite a loss of spatial and temporal coherence—which is inevitable when moving from a high-brightness synchrotron to a compact light source—XV data can still be extracted from the resulting images. In the present study, the same laboratory-based X-ray source was used to perform XV on a cohort of β-ENaC mice, a model of CF-like lung disease^26^. This data was then used to develop novel numerical methods that delineate symptoms of this disease. The success of XV lies in its ability to draw reliable and meaningful quantitative measures, and this study shows how this can be accomplished. In the future these techniques can be expected to be applied to the numerical characterization of CF lung disease in larger cohorts and other CF animal models. These methods allow analyses to be applied in a straightforward fashion and with minimal manual processing, to enable ongoing study and development of the treatment of CF and other respiratory diseases.

## Results and analysis

Here methods for extracting quantitative measures for lung health from the XV data are presented. These can be readily applied to large datasets with minimal manual intervention.

### Regional distribution of lung function

**Figure 1:**
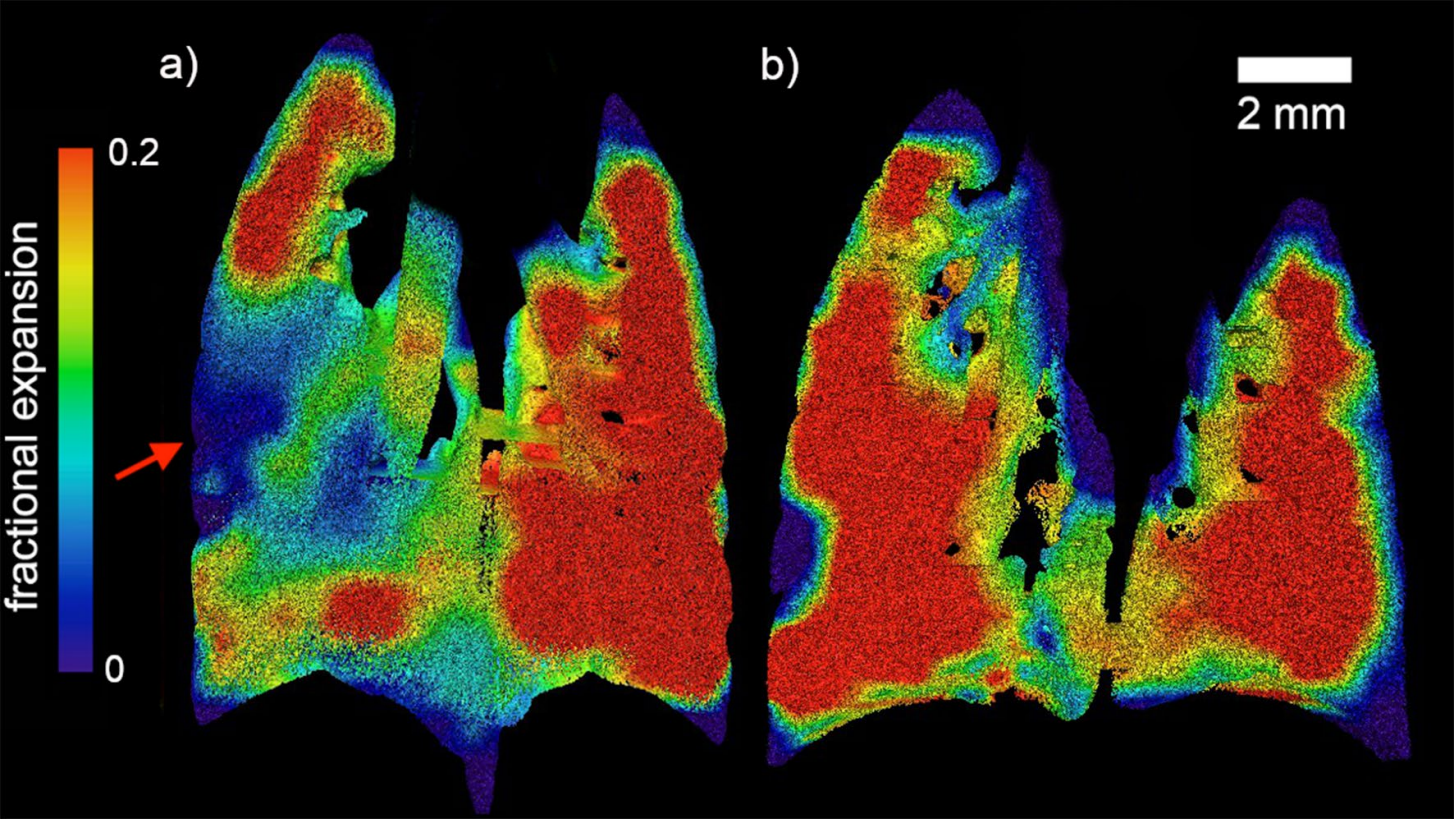
Regional fractional expansion of the lung since the beginning of the breath is characterized as a fraction of the XV voxel. Coronal slices of the expansion at peak inspiration are shown for (**a**) a β-ENaC mouse (M3) and (**b**) a healthy littermate (M9). The red arrow indicates a large region with reduced expansion in the lungs of the β-ENaC mouse. This image, and all images like it in this paper, are generated by superimposing 3D fractional expansion data onto a 3D rendered volume of the lungs, before taking a coronal slice of the now pseudo-coloured volume (Avizo, ThermoFisher Scientific).

Figure 1 maps the regional expansion of the lungs at the peak of the breath for both a β-ENaC mouse and its healthy littermate, as measured by XV. The expansion of each region of interest (ROI), defined by the XV voxel, is given as a fractional increase over the course of the breath, i.e. (change in volume of ROI)/(volume of ROI). The resulting measurement, *fractional expansion*, is a unitless quantity.

**Figure 2:**
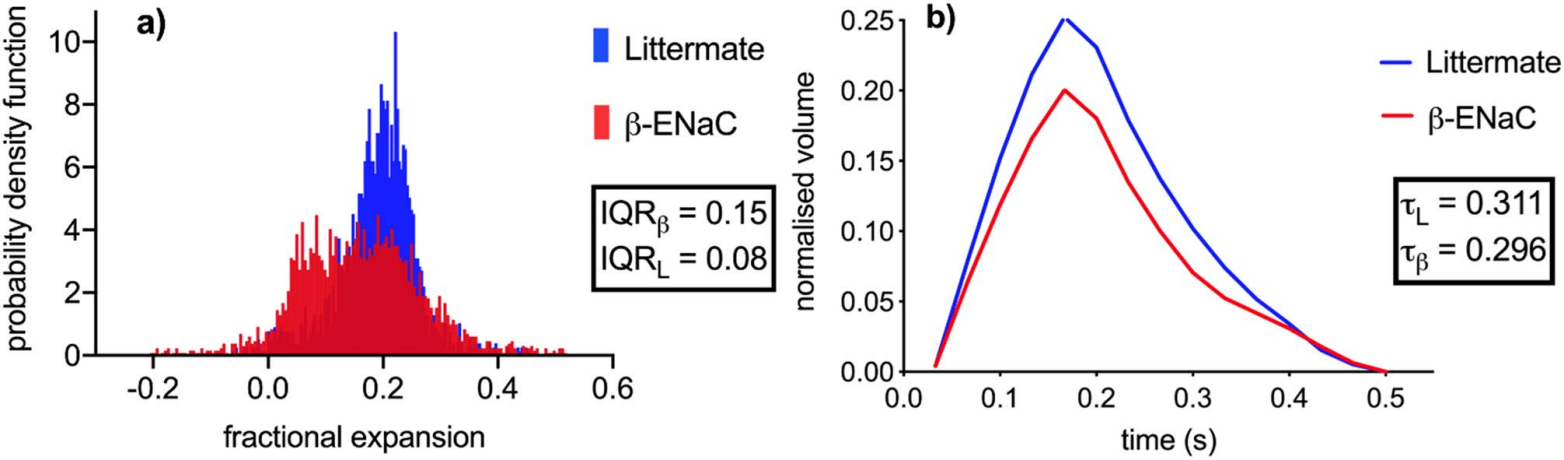
Comparative lung expansion analysis for a β-ENaC (M3, red) and littermate (M9, blue) pair of mice. (**a**) A histogram analysis of the fractional tissue displacement for the littermate and β-ENaC mice from Fig. 1, with the interquartile ranges of the normalised histogram shown as IQR. Note that Fig. 1 shows a single coronal slice, whereas the histogram is calculated from the entire volume. The fractional expansion for the β-ENaC mouse has a split peak. (**b**) The volume/time curve over the course of the ~ 0.5 s breath for the entire lung provides a global measurement of lung health. The volume of air breathed throughout the breath is expressed as a fraction of the entire lung volume; fractional volume. The healthy mouse breathes more air relative to the size of its lung. From this we can calculate the global expiratory time constant (τ).

The XV expansion data shown in Fig. 1 clearly allows the location of the airflow deficits to be determined within this plane inside the lung (see red arrow in panel 1a). In order to quantify differences throughout the entire lung volume, methods of calculating the distribution of tissue expansion have been developed here. An example of this approach is shown in Fig. 2a, which shows a histogram calculated from the fractional tissue displacement across the volume of the lung for each mouse shown in Fig. 1. The measurements for the β-ENaC mouse (from Fig. 1a) are shown in red and its healthy littermate (from Fig. 1b) in blue. The interquartile ranges (IQR) of each histogram are indicated in the graph. To adjust for variations in lung size across the cohort, the area under each histogram has been normalised to 1. In a homogeneously ventilated lung, the range of values for fractional tissue displacement should be narrow. In circumstances where there is heterogeneity due to a ‘patchy’ disease such as cystic fibrosis, a wider range of values (and therefore a higher IQR) is expected due to varied areas of poor ventilation and air trapping from mucus obstruction, as shown in Fig. 2a. Also apparent in the histogram from this particular β-ENaC mouse is the bimodal peak, where the lower peak represents regions of poor lung health (airflow), as indicated by the red arrow in Fig. 1a. This is likely to be caused by the presence of mucus obstruction which is a feature of this animal model^27, 28^. Our previous synchrotron-based study^24^ used histological sections to confirm that areas of reduced ventilation as measured by by XV analysis corresponded to mucus blockages in the bronchial tree^24^.

Concurrently, the global (total) expansion of the lung at each point in the breath was calculated in order to demonstrate the superiority of XV in its ability to produce information about the spatial distribution of lung function at any point in the breath, rather than a single global measure of lung function averaged across the entire breath. The global expiratory time constant (τ) (defined previously in^24^), is calculated as the time taken for 67% $(\sim 1/\sqrt{2}$) of the air to be expired from the lungs. The volume in Fig. 2b is calculated from the total magnitude of the 3D tissue displacement vectors over the entire lung for each point in time, and is normalised according to the total lung volume, which was calculated by evaluating the volume of the mask used for tissue segmentation (see “Image processing”). The purpose of this normalisation is to be able to express the volume of air breathed as a fraction of the total lung volume. In this example, the β-ENaC mouse has a fractional tidal volume that is lower than its healthy counterpart due to poorer expansion in some parts of the lung. Note that the measurements in Fig. 2b are expressions of the average health across the whole lung, without reference to the local distribution across the lung, and is analogous to measures such as FEV_1_. In contrast, the fractional expansion histogram (Fig. 2a) contains many spatially-separated measurements for the lung at the peak of the breath and is designed to visualise the airflow heterogeneity in the presence of this muco-obstructive disease.

### Quantifying distribution of disease

**Figure 3:**
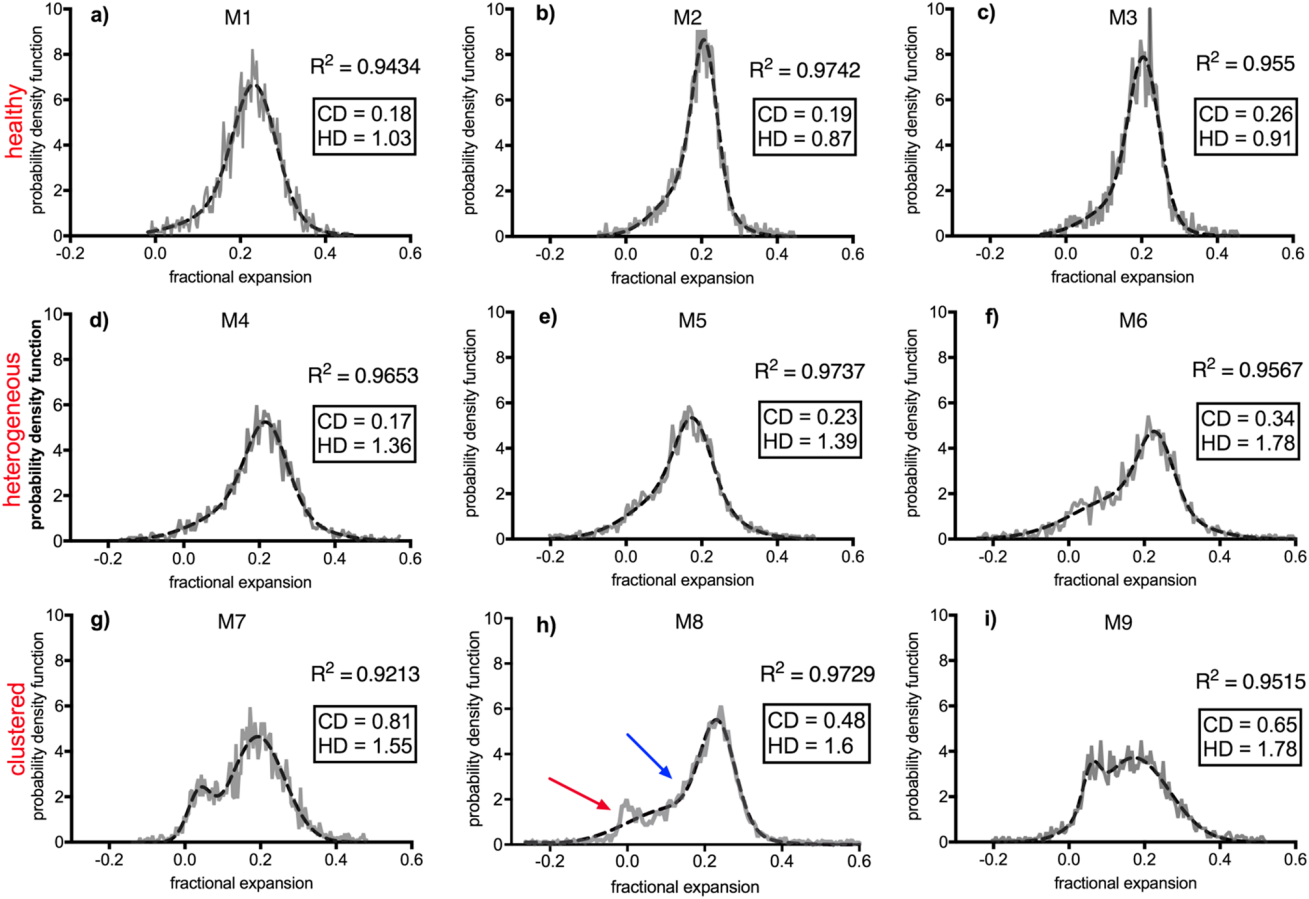
(**a**–**c**) show histograms from healthy littermates and (**d**–**i**) are those from β-ENaC mice. On each graph; the grey lines form the point along the histogram of the fraction expansion data and the broken black line represents the least squares double-Gaussian fit for the data. The goodness-of-fit is shown as R^2^. The extent of heterogeneous and clustered diseased is represented by HD and CD respectively, where a low CD and an HD value close to 1 indicates a healthy profile. In (**h**), the separate mode is indicated by the red arrow in addition to the bulge on the side of the main mode (blue arrow). The double gaussian fit summarises both deviations into a single curve.

As with human CF lung disease, there can be substantial variability in the severity and location of muco-obstructive disease between individual β-ENaC mice. Various presentations of CF-like disease have been categorised using the expansion histograms generated from the XV tissue-displacement calculations, by assigning numerical quantities to characterise their shape. In the implementation presented here a simple least-squares fit for a Gaussian distribution was adopted, and the statistical moments of the fitted curve have been used to numerically characterise the profile of the histogram and relate them to symptoms of disease. Figure 3 shows the histogram of nine different animals each with a score for two properties that describe how lung airflow is distributed throughout the lung. The measured local expansion across the lung, which represents functional changes, may collect around certain values (clustered) or vary widely (heterogeneous), thus each mouse is scored for the presence of either heterogeneous disease (HD) and clustered disease (CD), as defined below. All of the plots show the histogram of the raw data in a grey unbroken line, with the black broken line showing the nonlinear regression line-of-best-fit calculated by applying a least squares approximation to a double-Gaussian curve. The goodness-of-fit is shown on each figure as R^2^. For this experiment, three categories of histogram profiles are seen:

- *Healthy* The healthy animal shown in Fig. 2a (blue histogram) shows a typical *tall and narrow* expansion histogram from a healthy mouse lung, showing expansion data that falls into a narrow range and which represents homogeneous expansion. Figure 3a–c show the expansion histograms from three healthy mice, showing the characteristic tall and narrow peak.
- *Heterogeneous disease (HD)* When CF-like disease is established across the lung, we expect XV to demonstrate characteristic heterogeneous lung function, with the disease presenting across the volume of the lung. In lieu of a tall and narrow peak where most of the lung expands evenly (by the same percentage), we expect a *low and wide* peak, with a larger range of values as some parts of the lung expand less than other parts. Each sample receives a score for patchiness, calculated using the term $IQR/\underset{\_}{IQ{R}_{L}}$ where $IQR$ is the interquartile range of the histogram and $\underset{\_}{IQ{R}_{L}}$ is the average interquartile range for the littermate population. A healthy lung will have a value of close to 1 (see Fig. 3a–c), with the score increasing as lung health becomes more heterogeneous. Figure 3d–f show profiles for heterogeneous disease, each with a low and wide peak and higher HD values. Figure 3f has a heterogeneity level of 1.78, or rather 178% of healthy lung function variation.
- *Clustered disease (CD)* If airways are partially obstructed with mucus, poor ventilation and air trapping in the regions of the lung that receive air via those obstructed airways may result. In the end stages of this disease this may result in permanent damage to those regions due to atelectasis or bacterial infections that have been brought on by the presence of mucus. Where there is mucus preventing ventilation to a region of the lung, this region is less healthy than the rest of the lung, resulting in a clustered disease presentation. In the histogram of the expansion data, this is typically expressed as *bimodality*, or a *split peak* (as there could be two or more distinct regions). The diseased mouse in Fig. 2a shows such a peak. In order to determine whether or not the histogram of some expansion data possessed a second peak and how distinct the split between the peaks was, in this implementation a double-Gaussian distribution was consistently fit to each histogram. It was then possible to provide a score for bimodality using the term (μ_2_–μ_1_)/μ_2_; where μ_1_ and μ_2_ are the values of the two modes, or rather the mean values of each peak in the double Gaussian distribution. Note that a double-Gaussian function was fitted to the data not because we assumed the data followed a normal distribution, but because the characteristics of a Gaussian distribution (smooth, tends to zero at $\pm \infty$) made it a suitable basis function, conveniently applied to our data to readily extract functional measures. Low levels of mucus plugging of the large airways are indicated by low CD quantities in Fig. 3a–c. Figure 3f shows levels of clustered disease approaching 0.34, where a second peak is beginning to separate itself from the central mode. Figure 3g–i show expansion histograms with distinct CD presentation. In Fig. 3h, the algorithm has failed to pick up on the peak that is indicated by the red arrow. This is likely because there are three different regions, not two. The middle region (blue arrow) is not well-separated from the central mode. While the label ‘clustered’ strictly refers to a grouping of expansion values within the histogram, it is typical that this is associated with a spatial grouping of low-expansion pixels within the lung image.

**Figure 4:**
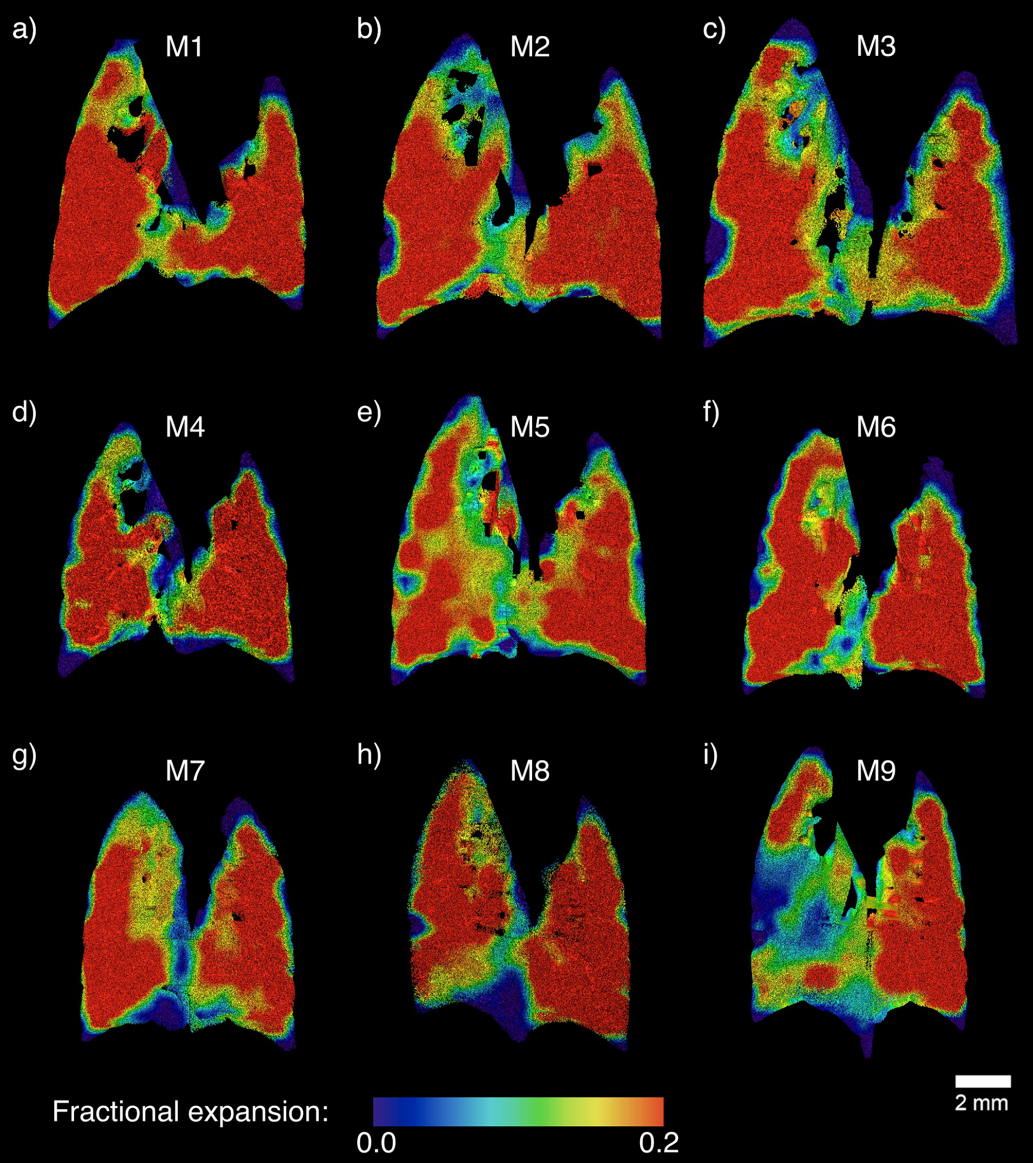
Coronal slices through the 3D expansion volumes that correspond to the histograms shown in Fig. 3, with the same mice shown in corresponding panels. Panels (**a**–**c**) show expansion maps from healthy littermates and (**d**–**i**) are those from β-ENaC mice. Note that a slice can reveal only a fraction of the whole volume, so does not necessarily reflect the level of disease heterogeneity that is apparent in Fig. 3.

Figure 4 shows coronal slices through the 3D expansion volumes that correspond to the histograms in Fig. 3.

**Figure 5:**
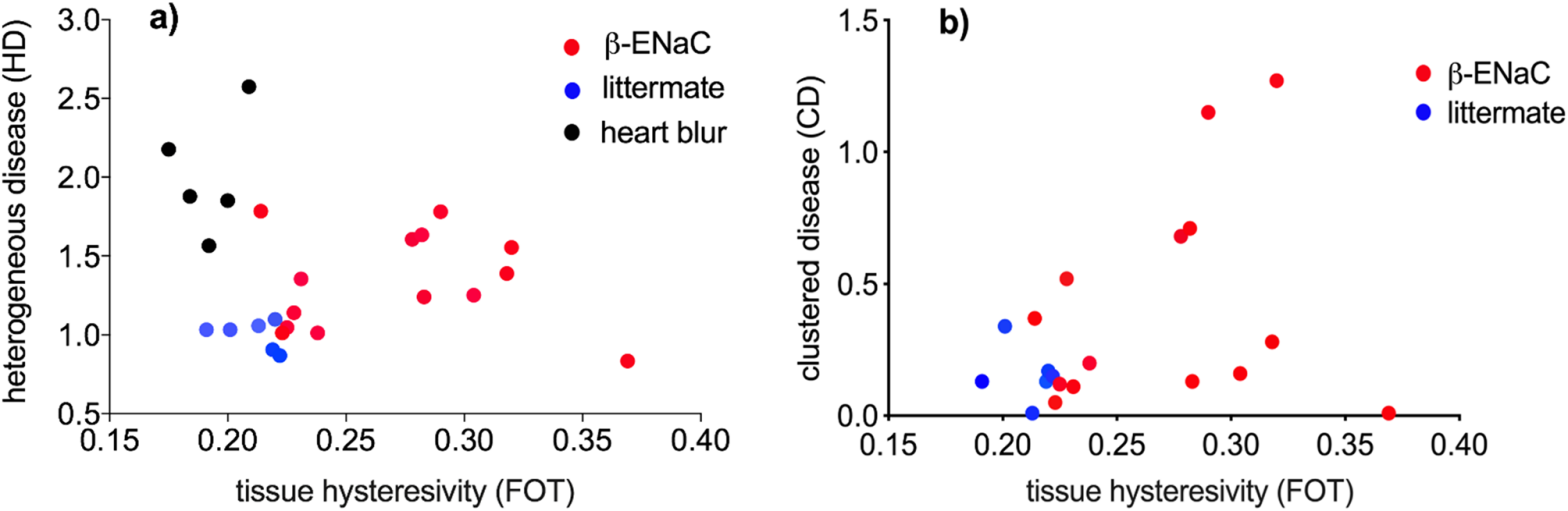
Plot of HD and CD against FOT results for tissue hysteresivity. We expect larger values for all quantities in animals with more severe disease, but we have shown the CF-like disease presents in a more complex manner than can be captured by a single quantity; while littermates are clustered closely together, β-ENaC points clearly display large variability in the lung disease present in these mice. (**a**) We have separated the images/samples we assessed to be suffering from heart blur (black) from the rest of the samples; (**b**) CD is not calculated for heart-blur samples.

In Fig. 5 the HD and CD values at the peak of the breath are plotted against the tissue hysteresivity (measured by FOT). In Fig. 5a, XV measurements that suffer from excessive “heart blur” (shown in black—this imaging artefact is described in the section below) are separated from the rest of the data points, which are themselves divided into β-ENaC (red) mice and their healthy littermates (blue). The blue data points consistently present with an HD index ~ 1, a homogeneous lung function presentation, while the red data points show greater variation, which is consistent with the range of disease presentation as exemplified earlier in the “Results and analysis” section. It is not expected that the full complexity of respiratory disease can be captured with a single measurement; nonetheless this simple plot shows a correlation with FOT measurements. Figure 5b further shows the large range of disease presentations that are seen within the group of β-ENaC mice, in this case characterised using the cluster disease index (CD). More variation is expected amongst samples with CF-like disease, due, for example, to the variability in symptoms discussed in “Results: quantifying symptoms of disease”.

### Heart blur

**Figure 6:**
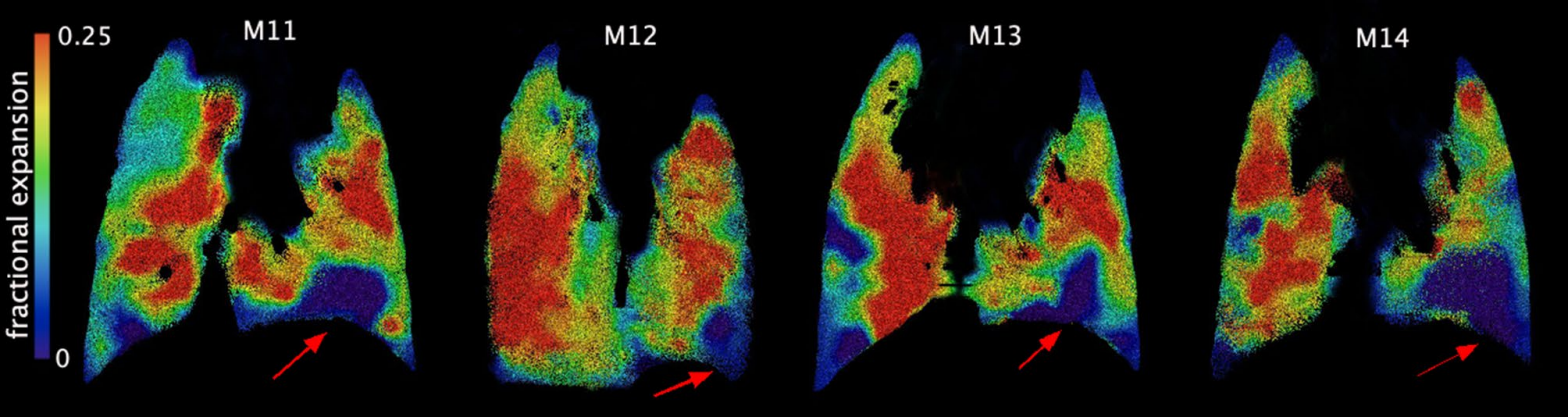
Heart blur throughout four different samples—all littermates—indicated by the red arrows. These areas record zero tissue displacement, as indicated by the colorbar at the left of the images.

As described in “Methods” the image acquisition was coordinated with ventilation. The heart, however, beats independently of image acquisition, resulting in some lung motion blur. Due to the spatial and temporal resolution of the laboratory-based source, along with the small exposure times required to acquire XV images, this blurring could cause local failure of the XV algorithm when it attempts to faithfully capture speckle motion. Figure 6 shows examples from four separate animals. Dubsky et al.^29^ used XV technology to show the manner in which cardiogenic oscillations affect airflow around the lung, by calculating the resulting tissue displacement. Their work showed that significant lung movement due to cardiac excursions is seen in the lower left regions of the lungs. This was also seen in our data, with the lower left region of the lungs (red arrows, Fig. 6) blurred in the CT image, preventing accurate measurement of tissue displacement using XV analysis. This resulted in unrealistic HD and CD values.

**Figure 7:**
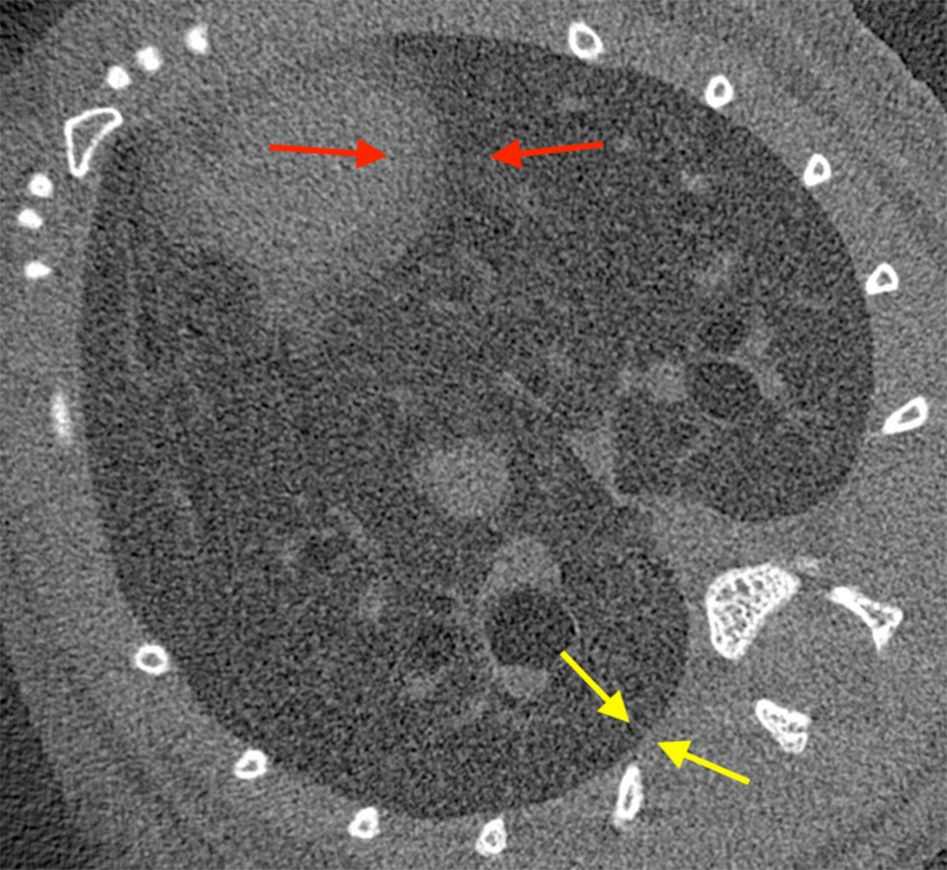
A single CT slice from the first frame of sample M11. The arrows indicate the distinction between a blurred edge (red) between the heart and lung, as a result of heart motion, and a well-defined edge (yellow), more distant from the heart.

A single CT slice from the first time point of the breath for mouse M11 above reveals this heart-motion associated blurriness (Fig. 7, red arrows). For comparison, the yellow arrows show a well-defined lung edge, situated away from the heart region.

The methods for detecting heart blur issues numerically are presented below in the “Discussion”.

## Discussion

This study successfully shows that XV—when performed on a laboratory source^25^—can capture and differentiate a range of lung disease presentations seen in β-ENaC mice via the novel quantification methods described here. Currently, clinically feasible methods for the quantification of regional lung airflows and heterogeneity are sparse, but this study shows that it is possible to measure the dynamics of muco-obstructive disease presentations in large groups of animals using XV without the need for a synchrotron X-ray source.

A significant outcome of this work is to provide the proof-of-principle for a straightforward means of numerically evaluating small animal lung health in the presence of symptoms of CF-like disease. It has been shown that the histogram for regional tissue displacement enabled by XV analysis provides information about the variability of airflow within the lung. Using the model described here, consisting of a score for both clustered disease (CD) and patchy/heterogeneous disease (HD), an overall presentation of CF-like disease can be quantified numerically. Combinations of these symptoms can account for a more rigorous approach to lung function evaluation than other techniques (e.g. a traditional lung function breath measurement such as spirometry, which measures breathing at the mouth and so averages airflow effects over the whole lung). When compared to FOT measurements for tissue hysteresivity, the HD score shows similarly low values for healthy animals. For diseased animals, there is greater variation—a complexity of presentation that can not be captured by a single measurement.

In future studies this scoring system should be suited to analysis of the accuracy of this system in separating disease states in larger cohorts, by using Machine Learning analysis of clustering and association of data points (as with^30^). Since the HD score is normalised to the mean variance of the healthy population, a new group of animals would have different baseline characteristics. Larger sample sizes would also enable us to use a Deep Learning model (see^31^) to determine the thresholds for the HD and CD which correspond to muoco-obstructive disease at its various stages. Future investigations can also determine the optimal combination of Gaussian curves which provides the best measure of CD. The use of more powerful statistical techniques such as functional data analysis for describing the shape of the histogram^32^ should also be investigated, along with an examination of how other respiratory conditions present using these numerical XV characterisations.

**Figure 8:**
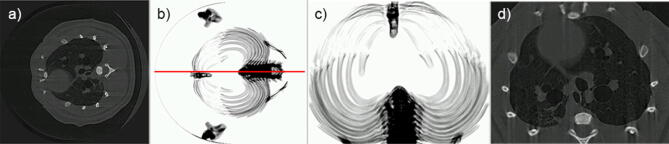
Mirror symmetry algorithm for automatic orientation and cropping. (**a**) A single slice from a CT reconstruction, pre-cropping. In (**b**), slices of the CT have been projected in the cranial/caudal direction to create a single image and the image thresholded to show the bones, revealing the symmetry of the ribcage. The red line indicates the line-of-best-symmetry as found by the algorithm, passing through the spine and sternum, allowing us to register their positions. (**c**) Using this result, the algorithm automatically corrects the orientation of the sample, and crops the image to the bones that act as a boundary for lung tissue; (**d**) shows a rotated and cropped slice*.*

The challenge of processing the volume of data that is required to develop this model presented here will be made easier with the inclusion of algorithms that can independently search data to detect symptoms of disease, without the need for user input. A number of image analysis algorithms are in development in order to accomplish this. For example, to orient each sample identically, we have implemented a *mirrored symmetry* approach on a CT image dataset that has been projected in the cranial/caudal direction to find the position of the spine, as shown in Fig. 8. The symmetry of the image is evaluated by comparing the features of the image with those of its reflection^33, 34^. Ultimately, the image is rotated to a position whereby the spine lies at the bottom of the image, to facilitate automated cropping. Correcting orientation provides the means to associate data points with their physical location across the lung, allowing, for example, automatic identification of heart blur due to characteristic proximity to the heart^29^.

Manual segmentation of the conducting airways or lung lobes, a preliminary step to XV, is time-consuming. Although providing impressive visualisation of lung function and highly accurate localisation of obstruction results, the inclusion of a manual step is not practical for large datasets. As a result, elements in image processing have been established that are designed to automatically segment the lungs from surrounding tissue, using thresholding, 3D morphological filters and continuity checks between slices. This approach will allow for the analysis of XV datasets to move from a new technique requiring some analysis training and effort into a highly accessible technique that can routinely evaluate large sets of data.

To address the challenge of heart blur, Lovric et al*.*^35^ have implemented a heartbeat-triggering gating technique into their image acquisition, although this is likely to be particularly challenging at the very high frame rates required for acquiring XV images at high ventilation rates. While experimental parameters have been optimised with the set-up for this experiment^36^, using a smaller spot size and higher power for the X-ray source, or a more sensitive detector, can increase the phase signal and reduce the amount of noise and blurriness in the X-ray images to improve our capabilities.

Translating XV to a laboratory-based X-ray source creates challenges associated with lower spatial resolution than what is available with a synchrotron-based source. However, the use of magnification at the laboratory X-ray source enables the use of large, highly-efficient pixels that can reduce the associated radiation dose, which is a key step on the translational path to the clinic. Rather than imaging the fine structures of the lungs at the very high resolution (and hence high dose) required to directly observe the presence of disease, disease can be inferred by analysing the expansion maps produced by XV. The subject can consequently be exposed to lower amounts of ionising radiation than would be required if these blockages were to be resolved directly. Techniques such as FOT and FEV_1_ provide single measures of lung function that are difficult to interpret alone, such that lung CT is often required to identify the structural abnormalities that might be the source of the change in lung function. The XV analysis technique presented here provides a more regional analysis of function across the lung and throughout the breath and provides the researcher, uniquely, the locations of airflow dysfunction Thus, XV has the potential to become a routine diagnostic tool to measure and monitor animal models, and ultimately humans, for improvements or declines in lung health. To translate XV to human use we have tomosynthesis experiments, large animal studies, and human clinical trials underway. Finally, XV will have applications beyond CF lung disease, and have value in other respiratory diseases such as asthma, COPD, emphysema and lung cancer, and in the development and assessment of respiratory therapeutics.

## Conclusions

Here, laboratory-based XV has been applied to the evaluation of lung disease heterogeneity in a group of β-ENaC mice and their healthy littermates. We also present a novel, straightforward and intuitive method for quantifying the distribution of their muco-obstructive disease. Future automated approaches will allow the application of this model to large sets of data in order to observe lung function changes during treatment, to develop a robust numerical model for CF lung disease. The combination of X-ray velocimetry and progressive automation of the data analysis is an important step in the development of more sophisticated methods of lung function testing, and should assist research internationally to improve the health and lives of people with cystic fibrosis, and a range of other lung diseases.

## Methods

### Image acquisition

**Figure 9:**
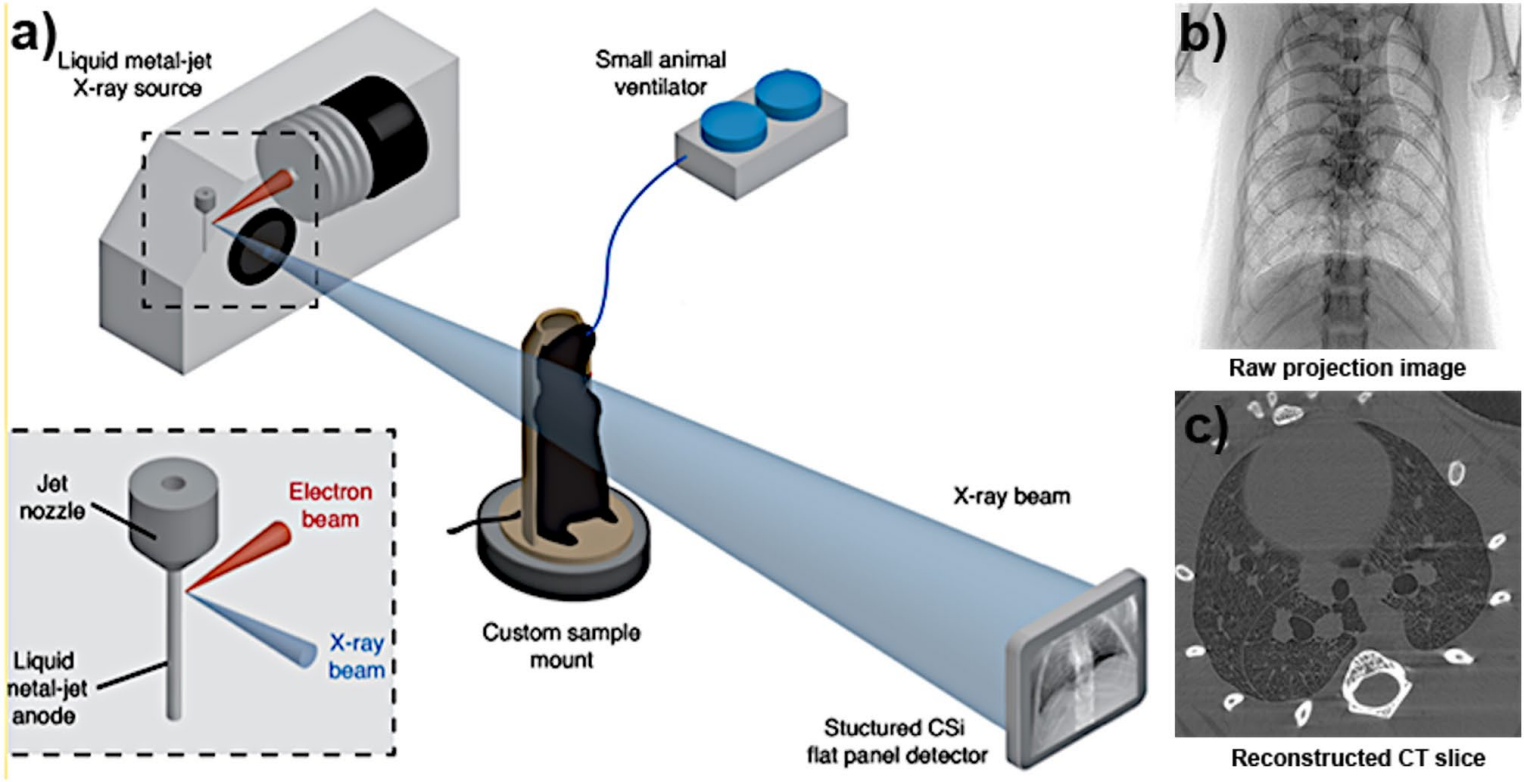
A diagram of the image acquisition process. (**a**) Experimental schematic: The mouse is mounted on a rotation stage in front of the x-ray beam, generated by an electron beam hitting the liquid–metal jet anode, pumped under high pressure to retain a laminar flow. A small-animal ventilator ensures that the detector acquires images at a rate that is synchronized to the breathing pattern of the mouse. The vacuum tube (not shown) lies between the mouse and the detector; (**b**) An example of a single projection image collected by the detector as the animal is rotated in front of it; (**c**) A single slice from the CT reconstruction of the projections from a single time point (Adapted from^25^).

All images were acquired at the Laboratory for Dynamic Imaging at Monash University on a propagation-based PCXI set-up shown in Fig. 9, with the X-ray beam (Excillum D2+, Excillum AB, Kista, Sweden) produced by an electron beam striking a liquid–metal anode. A high power (265 W) was used over a small source (spot size: 60 μm × 15 μm) to generate the flux needed to achieve an imaging rate of 30 frames per second, and coherence sufficient to generate the phase contrast necessary across the lung volume^37^. With a conventional solid-metal anode, one would have to take care not to overheat the target while attempting to generate a higher flux. However, by using a liquid–metal-jet anode—pumped under high pressure to maintain a laminar flow—there was no concern of approaching the limits of heating the metal target in order to extract sufficiently high flux.

The source-to-detector distance was fixed at 3,363 mm, with a maximum source-to-sample distance of 467 mm. The translation stage enabled the mice to be moved toward and away from the source to alter the zoom factor as required. To produce phase contrast in the resulting images, a minimum propagation (sample to detector) distance of 2,896 mm (through the ~ 30 cm diameter vacuum tube) was used. To minimise scattering and avoid an associated reduction in image contrast, the x-rays were propagated through a vacuum tube before reaching the detector.

### Animal experiments

All experiments were approved by the Monash University Animal Ethics Committee and conformed to the guidelines set out in the NHMRC Australian Code of Practice for the Care and Use of Animals for Scientific Purposes.

β-ENaC mice (n = 15), aged 45–84 days (median = 62 days) at the time of imaging, were used for all experiments^26^. Mice were bred on a C57Bl/6N background, and supplied from our specific pathogen-free breeding colony (Monash Animal Research Platform). Littermate controls (n = 10) were used to minimise the effects of strain on the lung phenotype. Offspring were genotyped at 3 weeks of age via PCR of genomic DNA as previously described^26^.

Mice were anaesthetised with an intraperitoneal (i.p) injection of a 10 μl/g body weight mixture of medetomidine (0.1 mg/ml, Orion Corporation, Finland) and ketamine (7.6 mg/ml, Parnell Laboratories, Australia), and surgically intubated. The endotracheal tube was attached to a custom-built small animal pressure-controlled ventilator (AcuVent, Notting Hill Devices, Australia) at 12 cmH_2_O PIP and 2 cmH_2_O PEEP with a respiratory rate of 120 breaths per minute (inspiration time of 0.15 s and an expiration time of 0.35 s). To maintain the normal dynamics of lung function, a paralytic was not used.

Mice were mounted in a vertical position in front of the source on a custom high-precision rotation stage (Zaber Technologies, Vancouver, Canada). Mice were rotated through 360 degrees at 1.5 degrees per second while a flat-panel detector (PaxScan, Varian Medical Systems, Palo Alto, CA, USA) captured images at the rate of 30 Hz to acquire a total of 7,200 images per mouse. Image acquisition was triggered by the ventilator, and was gated to collect 15 images throughout the breathing cycle. Airway pressure and flow were monitored throughout the experiments.

At the completion of the imaging experiments, global lung mechanics were measured using a modification of the forced oscillation technique (FOT). Mice were hyperventilated at 400 breaths per minute for 60 s to induce brief (6 s) periods of apnea. During apnea, an oscillatory signal, generated by a loudspeaker, containing 9 frequencies ranging from 4–38 Hz was introduced into the tracheal cannula via a wavetube of known impedance. The impedance of the respiratory system (Zrs) was calculated. A four-parameter model with constant phase tissue impedance^38^ was fit to the data to Zrs spectrum allowing determination of tissue hysteresivity which is calculated as the ratio of the tissue damping to tissue elastance^39^.

### Image processing

To complete the XV cross-correlation analysis, the 7,200 projections were organised, or *binned*, into their time points resulting in 400 projections per time point, with a total of 15 time points across the 500 ms breathing period. Computed tomographic reconstruction was performed for each set of projections giving 15 separate CT reconstructions, one for each of the 15 stages of the breath. Each CT consisted of 1,024 slices, each 1,024 pixels by 1,024 pixels. The effective voxel size varied from mouse-to-mouse.

Using *Avizo* software (ThermoFisher Scientific), the CT volume representing the beginning of the breath was used to create a mask for isolating the lung tissue from the rest of the animal. The volume of the mask was also used to determine the total voxel size of the lung, which, after accounting for variation in effective voxel size, was used to normalise the volumetric results.

For XV, an interrogation region size of 64 ⨉ 64 ⨉ 64 pixels with an overlap of 50% between successive interrogation windows were used, producing a XV voxel size of 32 ⨉ 32 ⨉ 32 pixels. The XV output showed the magnitude and direction of the lung tissue motion vectors between each time point. This displacement of tissue denotes lung expansion, and was expressed in voxels per frame. The mainstem bronchi were removed from the expansion map images to improve clarity.

## Data Availability

The data that support the findings of this study are available on reasonable request from the corresponding authors. The XV analysis code that supports the findings in this study is not publicly available due to patent restrictions. Code may however be available from the authors upon reasonable request and with permission of Monash University and 4Dx Limited.
